# Clinical Manifestations Vary with Different Age Spectrums in Infants with Kawasaki Disease

**DOI:** 10.1100/2012/210382

**Published:** 2012-02-15

**Authors:** Hao-Chuan Liu, Chiao-Wei Lo, Betau Hwang, Pi-Chang Lee

**Affiliations:** ^1^Department of Pediatrics, Taipei Veterans General Hospital Yuanshan Branch, Yilan 264, Taiwan; ^2^Department of Pediatrics, National Yang-Ming University Hospital, Yilan 260, Taiwan; ^3^Taipei City Hospital Zhongxiao Branch, Taipei 115, Taiwan; ^4^Department of Pediatrics, Taipei Veterans General Hospital and National Yang-Ming University, Taipei 112, Taiwan

## Abstract

*Background*. Kawasaki disease (KD) is an acute systemic vasculitis with unknown etiology. The diagnosis of KD depends on clinical manifestations. The prevalence of coronary artery abnormality (CAA) is 11.0% and results in cardiac sequelae, such as myocardial infarction or coronary aneurysm, which are the most serious complications in KD. *Methods*. We divided KD's children into different age groups: ≤6 months old, 7 months to 1 year old, and >1 year old, respectively. Different parameters were compared in each group. *Results*. Infants ≤6 months old are less likely to fulfill KD's major diagnostic criteria within 10 days, are prone to develop incomplete KD with the lowest cholesterol level, and have the greatest chance to have CAA and the laboratory features associated with CAA, such as the longest time needed to confirm CA diagnosis, lower hemoglobin level, lower albumin level, and higher platelet count. Infants <1 year old develop higher percentage of leukocytosis and sterile pyuria. But this group has fewer patients with neck lymphadenopathy.

## 1. Introduction

Kawasaki disease (KD), or mucocutaneous lymph node syndrome, is an acute febrile illness of young children. About 80% of the cases aged between 6 month to 5 years [1]. Male is predominant with the male to female ratio about 1.5 : 1 [2]. The incidence of KD varies around the world. It is 20 times more common in North East Asia. The highest rate was reported in Japan as 218.6 per 100,000 children from 0 to 4 years of age in the year 2008, 69 per 100,000 children under 5 years old in Taiwan, 86.4 per 100,000 in Korea, 20.8 per 100,000 in the USA, and 8.39 per 100,000 in England. The annual survey of KD shows the incidence rate increases year by year [3–7]. 

The prevalence of coronary artery abnormality (CAA) during the KD's acute phase was 11.0% in the 20th nationwide survey [4]. Previous literatures indicated that, among patients of KD, young infants have increased risks of CAA. They usually suffer from prolonged fever without classic KD's presentation [8–11]. This may make the diagnosis challenging and delay intravenous immunoglobulin treatment. Such delay causes higher probability of developing CAA [12]. The cutoff points of infant's age in previous studies were 6 months or 1 year old [8–11, 13, 14]. Both of the age spectrums revealed similar results. In our study, we further subdivide infant age into ≤6 months old and 7 months to 1 year old. With those older than 1 year old, we compared the differences of clinical manifestations, laboratory results, and echocardiography results between each group.

## 2. Methods

A retrospective chart review for all children with the diagnosis of KD admitted to the Taipei Veterans General Hospital from January 1993 to March 2008 was conducted. All the patients were divided into three groups on the basis of the age of KD at diagnosis: group 1 (≤6 months old), group 2 (7~12 months old), and group 3 (>1 year old). All the clinical manifestations, laboratory results, and echocardiography findings were recorded.

The diagnosis of complete KD was based on fever for more than 5 days together with ≥4 of the 5 major clinical manifestations, which included change in extremities, polymorphous exanthema, bilateral painless bulbar conjunctiva injection without exudate, change in lip and oral cavity, and cervical lymphadenopathy larger than 1.5 cm in diameter. Patients who had fever with less than 4 characteristic manifestations were diagnosed as incomplete KD when coronary artery involvement was detected by two-dimensional echocardiography [2]. All the diagnoses of these patients were confirmed by more than 2 pediatric cardiologist, and other possible diseases were excluded.

Two-dimensional and color flow mapping echocardiography were performed in every patient at admission. Coronary artery abnormalities and the valves regurgitation were recorded and reviewed. If coronary artery had perivascular brightness under echocardiography, coronary artery involvement was defined [15]. Coronary artery dilatation's definition was according to Japanese Ministry of Health's diagnostic criteria of coronary artery lesions in KD: the coronary artery internal diameter >3 mm in children <5 years old, the coronary artery internal diameter >4 mm in children ≥5 years old, and the internal diameter of a segment ≥1.5 times of the adjacent segment [16]. Coronary aneurysm was defined as coronary artery internal diameter ≥5 mm, regardless of age [17].

Continuous data were analyzed statistically by one-way ANOVA test followed by post-hoc Holm-Sidak test for pairwise comparison. Categorical data were compared with each other statistically by Chi-square analysis with Yates correction for continuity or Fisher's exact test while the sample size was small. Statistical significant was defined as *P* < 0.05.

## 3. Results

A total of 145 patients were identified. Group 1 (≤6 months old), group 2 (7–12 months old), and group 3 (>1 year old) included 30 (21%), 35 (24%), and 80 (55%) infants, respectively. The collected data were compared in Table 1, which included days between disease onset to diagnosis, male to female ratio, diagnosis of complete KD or incomplete KD, fulfilling KD's major clinical criteria, and clinical manifestations. Infants younger than 6 months old took the longest time from the day of symptoms onset to diagnosis, and they had the smallest proportion of patients fulfilling complete diagnostic criteria of KD while at the same time the largest proportion fulfilling the criteria of incomplete KD. Infants younger than 12 months old were less common to develop neck lymphadenopathy but were more frequently to be documented with sterile pyuria. The ratio of female patients increased in children older than 1 year old, comparing to those less than 6 months old.

While comparing with laboratory results, patients who were <1 year old had higher white blood cell (WBC) count. Patients who were ≤6 months old had statistically the lowest hemoglobin, the lowest cholesterol, and the lowest albumin level, but the highest platelet count. The liver enzyme amount, triglyceride level, high-density lipoprotein level, and C-reactive protein (CRP) level were similar among the 3 groups (Table 2). The low-density lipoprotein (LDL) level was the highest in those older than 1 year old and was statistically significant comparing to those younger than 6 months old. Even within normal limit, there was a trend that LDL level grew higher with age. Echocardiography results showed similar coronary artery involvement, similar irregular coronary artery surface, and similar valve regurgitation between the 3 groups. Infants ≤6 months old were the most likely to develop coronary artery dilatations. Even not statistically significant between group 1 and 3, patients ≤6 months old had more coronary artery aneurysm (Table 3).

## 4. Discussion

Kawasaki disease is an acute febrile illness with systemic vasculitis that predominantly occurs in infant and young children. The etiology is still unknown but the epidemiology features suggest an infectious origin. Due to the absence of a specific diagnostic test, clinical criteria have been used to establish the diagnosis. Other clinical and laboratory findings such as leukocytosis, elevated CRP, diarrhea, cough, rhinorrhea, and perianal desquamation are also suggestive of the diagnosis [17]. The major complication of KD is CAA, which includes coronary artery dilatation and coronary aneurysm [17]. CAA may cause myocardial ischemia, myocardial infarction, or sudden death [18, 19]. Previous reports have shown that patients <1 year old are more likely to have incomplete KD and CAA [10, 11, 20]. In our retrospective study, we demonstrated that coronary artery dilatation and CAA-associated laboratory features are more common in infants younger than 6 months old. Those patients younger than 1 year old have more WBC count and more sterile pyuria, but less neck lymphadenopathy.

Demographic and laboratory factors were surveyed extensively in previous studies for identifying risk factors of CAA in KD. Demographic factors include male gender [21–23], race [22], longer time from fever to treatment [23–25], incomplete KD [26], and younger age [9, 10, 13, 20–22, 27]. Laboratory factors include lower hemoglobin level [25, 28, 29], higher platelet count [24, 30], higher WBC count [28, 29], lower serum albumin [31], and higher alanine aminotransferase [29]. The three most commonly reported laboratory factors were lower hemoglobin level, higher platelet count, and lower albumin level [9]. Although infants younger than 4 months old experienced physiologic anemia, infants in this study showed that those ≤6 months old had not only the least favorable laboratory results, but also the highest rate of coronary artery dilatation. Even not statistically significant between group 1 and 3, infants ≤6 months old were also seen to have the trend of much coronary artery giant aneurysm comparing to other age spectrums. In addition to the unfavorable laboratory results, in this study, infants ≤6 months had longer days between the disease onset to diagnosis, more incomplete KD, and fewer patients fulfilling the diagnosis criteria within 10 days, which were also risk factors of CAA [24–26, 32]. In our study, we demonstrated that infants ≤6 months were more vulnerable of having CAA in comparison to those <1 year old [8, 10, 13, 22, 31].

Besides that the risk factors of CAA are more in KD's infants younger than 6 months old, the difference of coronary artery anatomy by age might exacerbate the possibility of CAA. The pathogenesis of the vasculitis in KD had been proposed by several literatures. Jennette [33] described that the earliest lesion of CAA is subendothelial accumulation of leukocytes, including monocytes, macrophages, neutrophils, and T-lymphocytes [34]. The following transmural infiltration of mononuclear lymphocytes results in transmural inflammation as well as smooth muscle cell edema and degeneration in the vascular media. With the disease progress, the infiltration extends from the vascular media into the adventitia. Finally, the cascade of events result in destructions of the vascular media and aneurysm formation [35]. In human coronary artery, Ikari et al. [36] performed serial coronary artery sections for babies from 17-week gestation to 23 months after birth. They found that the coronary artery intima is rarely noted before 30-week gestation. Only 38% of the newborns have coronary artery intima in the first week of life with intima/media ratio nearly 0.1. Until three months after birth, vascular intima can be detected in all infants. This result gave us a hint that there might be a relationship between the delayed development of the vascular intima and the higher rate of CAA. The higher CAA incidence in infants ≤6 months old might be due to the loss of or a thin vascular intima in young infants. Large amount of the leukocytes may penetrate the endothelium and infiltrate into the media and the adventitia easier if there is no or a thin endothelium. Large amount of the infiltrations would cause more severe vascular inflammation with serious vascular medial destruction and aneurysm formation.

Patients with KD usually have peripheral leukocytosis. An elevated WBC count usually indicates an infection, inflammation, allergic disorder, or some forms of malignancy. In our study, patients <1 year old have statistically higher WBC count comparing to those older than 1 year of age. Multiple reports have indicated that an infectious agent is highly possibly the etiology of KD. Burns et al. [37] had suggested an environmental trigger in KD due to pronounced seasonality and temporal clustering of cases in Japan. Esper et al. [38] had found “New Haven Coronavirus” in KD patients' respiratory secretion. Rowley et al. [39] had used IgA antibody and detected a specific antigen within the coronary arteries, bronchial epithelium, and inflamed KD tissues. These results have been supporting the hypothesis that infection is the etiology of KD. The leukocytosis in KD might be due to the systemic reaction toward infection as well as acute inflammation of the coronary arteries. Focal inflammation of the coronary artery, such as coronary artery disease (CAD) in adult, can also cause leukocytosis [22, 24, 25]. In patients with KD, with the medium-sized elastic arteries inflammation, coronary artery inflammation should at least be part of the reason of leukocytosis.

Kawasaki disease is a male predominate disease [2]. The boys also have a higher risk of CAA in previous report [10]. In our study, we have found the trend of increasing incidence of female patients with age. However, the reason was unknown or might be due to our small sample size. Urinalysis revealed sterile pyuria in about 33% of the KD's patient. This was probably due to urethritis instead of bacterial urinary tract infection [17]. In our study, sterile pyuria is more frequent in patients <1 year than older children. That might indicate that infants younger than 1 year old suffer from more serious urethritis than children. We have also demonstrated that neck lymphadenopathy is the least common sign of the KD's five major clinical criteria in infants <1 year old. Therefore, echocardiography should be performed to evaluate coronary artery in infants who have prolonged fever with the suggestive KD's clinical signs and laboratory results.

There are several limitations in the present study. The case numbers are not large enough to represent general population. Furthermore, we conduct a retrospective study; there are some missing data which make the case numbers even smaller to be analyzed. Also, we cannot exclude the possibility that other infectious diseases which had similar clinical signs and symptoms were diagnosed as incomplete Kawasaki disease.

## 5. Conclusion

After subdividing the infant age, we find that infants younger than 6 months old take the longest time to diagnose, are the least to fulfill the clinical major criteria, and have the least favorable laboratory results which are the risk factors of developing CAA. They are also more likely to have incomplete KD and coronary artery dilatation. Coronary giant aneurysm, even not statistically significant, is also more frequently observed in younger infants. Instead, the WBC count, sterile pyuria, and neck lymphadenopathy are seen more often in those younger than 1 year old. 

## Figures and Tables

**Table 1:** Incidence and clinical manifestations.

Variable	Group 1 (*n* = 30)	Group 2 (*n* = 35)	Group 3 (*n* = 80)	*P* value		
				Group 1 versus group 2	Group 1 versus group 3	Group 2 versus group 3
Days between disease onset to diagnosis, mean ± SD	8.5 ± 3.7	6.3 ± 2.5	6.7 ± 3.1	0.006^†^	0.010^†^	0.506
M/F	24/6	26/9	45/35	0.803	0.038^†^	0.105
Fulfill diagnosis criteria in 10 days, *n* (%)	11 (37%)	23 (66%)	64 (80%)	0.037^†^	<0.001^†^	0.160
Incomplete Kawasaki disease, *n* (%)	19 (63%)	12 (34%)	16 (20%)	0.037^†^	<0.001^†^	0.160

Major criteria						

Fever > 5 days, *n* (%)	30 (100%)	33 (94%)	75 (94%)	0.945	0.375	0.754
Bilateral bulbar conjunctiva injection, *n* (%)	25 (83%)	35 (100%)	75 (94%)	0.017^†^	0.187	0.310
Change in mucosa of oropharynx, *n* (%)	26 (87%)	32 (91%)	73 (91%)	0.695	0.721	0.743
Change of the extremities, *n* (%)	12 (40%)	18 (51%)	40 (50%)	0.502	0.471	0.951
Skin rash, *n* (%)	27 (90%)	32 (91%)	73 (91%)	1.00	0.866	0.743
Lymphadenopathy, *n* (%)	4 (13%)	12 (34%)	51 (64%)	0.096	<0.001^†^	0.007^†^

Other clinical manifestations						

Upper respiratory tract infection, *n* (%)	18 (60%)	21 (60%)	35 (44%)	0.800	0.192	0.161
Diarrhea/vomiting, *n* (%)	12 (40%)	14 (40%)	19 (24%)	0.800	0.147	0.121
Sterile pyuria, *n* (%)	14 (40%)	14 (40%)	16 (20%)	0.772	0.011^†^	0.044^†^
Perianal desquamation, *n* (%)	7 (23%)	2 (6%)	16 (20%)	0.096	0.905	0.097

^†^Statistical significance.

**Table 2:** Laboratory results.

Variable	Group 1 (*n* = 30)	Group 2 (*n* = 35)	Group 3 (*n* = 80)	*P* value		
				Group 1 versus group 2	Group 1 versus group 3	Group 2 versus group 3
WBC, mean ± SD, (/cumm)	19810.0 ± 10880.3	17242.9 ± 5830.0	13653.1 ± 4826.1	0.127	<0.001^†^	0.009^†^
Hemoglobin, mean ± SD, (g/dL)	9.9 ± 1.2	10.7 ± 1.6	11.0 ± 1.2	0.015^†^	<0.001^†^	0.199
Platelet, mean ± SD, (/cumm)	492966.7 ± 163522.1	397200.0 ± 140359.0	355337.5 ± 132276.4	0.007^†^	<0.001^†^	0.145
CRP, mean ± SD, (mg/dL)	11.0 ± 7.1	9.4 ± 5.9	9.8 ± 6.9	NS		
Aspartate aminotransferase, mediam (range), (units/L)	37 (12~256) (*n* = 26)	23 (16~191) (*n* = 32)	27.5 (17~277) (*n* = 73)	NS		
Alanine aminotransferase, mediam (range), (units/L)	26.5 (9~235) (*n* = 27)	34 (12~639) (*n* = 32)	29 (9~525) (*n* = 75)	NS		
Cholesterol, mean ± SD, (mg/dL)	102.0 ± 26.1 (*n* = 22)	124.8 ± 29.0 (*n* = 29)	136.3 ± 31.8 (*n* = 68)	0.008^†^	<0.001^†^	0.089
Triglyceride, mean ± SD, (mg/dL)	121.1 ± 51.0 (*n* = 17)	139.8 ± 67.6 (*n* = 30)	124.2 ± 49.1 (*n* = 69)	NS		
HDL, mean ± SD, (mg/dL)	21.4 ± 9.5 (*n* = 17)	22.0 ± 13.5 (*n* = 27)	25.0 ± 10.3 (*n* = 62)	NS		
LDL, mean ± SD, (mg/dL)	58.2 ± 21.2 (*n* = 17)	73.2 ± 26.2 (*n* = 27)	82.2 ± 28.1 (*n* = 60)	0.071	0.001^†^	0.150
Albumin, mean ± SD, (g/dL)	3.4 ± 0.4 (*n* = 15)	3.8 ± 0.5 (*n* = 26)	3.8 ± 0.5 (*n* = 63)	0.026^†^	0.015^†^	0.925

^†^Statistical significance.

CRP: C-reactive protein; HDL: high-density lipoprotein; LDL: low-density lipoprotein; NS: not statistically significant difference between groups; WBC: white blood cell.

**Table 3:** Echocardiography results.

Variable	Group 1 (*n* = 30)	Group 2 (*n* = 35)	Group 3 (*n* = 80)	*P* value		
				Group 1 versus group 2	Group 1 versus group 3	Group 2 versus group 3
Coronary artery involvement, *n* (%)	18 (60%)	20 (57%)	36 (45%)	0.985	0.235	0.319
Coronary artery dilatation, *n* (%)	14 (47%)	6 (17%)	17 (21%)	0.021^†^	0.016^†^	0.800
Irregular coronary artery surface, *n* (%)	16 (53%)	16 (46%)	34 (43%)	0.716	0.423	0.908
Coronary artery giant aneurysm, *n* (%)	8 (27%)	1 (3%)	8 (10%)	0.009^†^	0.057	0.35
Valve regurgitation, *n* (%)	15 (50%)	15 (43%)	31 (39%)	0.744	0.396	0.836

^†^Statistical significance.

## References

[1] Harnden A, Takahashi M, Burgner D (2009). Kawasaki disease. *BMJ*.

[2] (2001). Diagnostic guidelines for Kawasaki disease. *Circulation*.

[3] Huang WC, Huang LM, Chang IS (2009). Epidemiologic features of Kawasaki disease in Taiwan, 2003–2006. *Pediatrics*.

[4] Nakamura Y, Yashiro M, Uehara R (2010). Epidemiologic features of Kawasaki disease in Japan: results of the 2007-2008 nationwide survey. *Journal of Epidemiology*.

[5] Park YW, Han JW, Park IS (2005). Epidemiologic picture of Kawasaki disease in Korea, 2000–2002. *Pediatrics International*.

[6] Harnden A, Mayon-White R, Perera R, Yeates D, Goldacre M, Burgner D (2009). Kawasaki disease in England: ethnicity, deprivation, and respiratory pathogens. *Pediatric Infectious Disease Journal*.

[7] Holman RC, Belay ED, Christensen KY, Folkema AM, Steiner CA, Schonberger LB (2010). Hospitalizations for Kawasaki syndrome among children in the United States, 1997–2007. *Pediatric Infectious Disease Journal*.

[8] Tseng CF, Fu YC, Fu LS, Hwang B, Chi CS (2001). Clinical spectrum of Kawasaki disease in infants. *Zhonghua Yi Xue Za Zhi*.

[9] Manlhiot C, Yeung RS, Clarizia NA, Chahal N, McCrindle BW (2009). Kawasaki disease at the extremes of the age spectrum. *Pediatrics*.

[10] Rosenfeld EA, Corydon KE, Shulman ST, Mason W, Takahashi M, Kuroda C (1995). Kawasaki disease in infants less than one year of age. *Journal of Pediatrics*.

[11] Burns JC, Wiggins JW, Toews WH (1986). Clinical spectrum of Kawasaki disease in infants younger than 6 months of age. *Journal of Pediatrics*.

[12] Anderson MS, Todd JK, Glodé MP (2005). Delayed diagnosis of Kawasaki syndrome: an analysis of the problem. *Pediatrics*.

[13] Moreno N, Mendez-Echevarria A, de Inocencio J (2008). Coronary involvement in infants with Kawasaki disease treated with intravenous gamma-globulin. *Pediatric Cardiology*.

[14] Chang FY, Hwang B, Chen SJ, Lee PC, Meng CCL, Lu JH (2006). Characteristics of Kawasaki disease in infants younger than six months of age. *Pediatric Infectious Disease Journal*.

[15] Newburger JW, Taubert KA, Shulman ST (2003). Summary and abstracts of the Seventh International Kawasaki Disease Symposium: december 4–7, 2001, Hakone, Japan. *Pediatric Research*.

[16] Research Committee on Kawasaki Disease (1984). *Report of Subcommittee on Standardization of Diagnostic Criteria and Reporting of Coronary Artery Lesions in Kawasaki Disease.*.

[17] Newburger JW, Takahashi M, Gerber MA (2004). Diagnosis, treatment, and long-term management of Kawasaki disease: a statement for health professionals from the committee on rheumatic fever, endocarditis, and Kawasaki disease, council on cardiovascular disease in the young, American Heart Association. *Pediatrics*.

[18] Kato H, Sugimura T, Akagi T (1996). Long-term consequences of Kawasaki disease. A 10- to 21-year follow-up study of 594 patients. *Circulation*.

[19] Dajani AS, Taubert KA, Gerber MA (1993). Diagnosis and therapy of Kawasaki disease in children. *Circulation*.

[20] Chuang CH, Hsiao MH, Chiu CH, Huang YC, Lin TY (2006). Kawasaki disease in infants three months of age or younger. *Journal of Microbiology, Immunology and Infection*.

[21] Nakamura Y, Fujita Y, Nagai M (1991). Cardiac sequelae of Kawasaki disease in Japan: statistical analysis. *Pediatrics*.

[22] Belay ED, Maddox RA, Holman RC, Curns AT, Ballah K, Schonberger LB (2006). Kawasaki syndrome and risk factors for coronary artery abnormalities: United States, 1994–2003. *Pediatric Infectious Disease Journal*.

[23] Zhao LL, Wang YB, Suo L (2011). Meta-analysis of the risk factors for coronary artery lesion secondary to Kawasaki disease in Chinese children. *Zhonghua Er Ke Za Zhi*.

[24] McCrindle BW, Li JS, Minich LL (2007). Coronary artery involvement in children with Kawasaki disease: risk factors from analysis of serial normalized measurements. *Circulation*.

[25] Morikawa Y, Ohashi Y, Harada K (2000). Coronary risks after high-dose *γ*-globulin in children with Kawasaki disease. *Pediatrics International*.

[26] Rowley AH, Gonzalez-Crussi F, Gidding SS (1987). Incomplete Kawasaki disease with coronary artery involvement. *Journal of Pediatrics*.

[27] Shulman ST, McAuley JB, Pachman LM (1987). Risk of coronary abnormalities due to Kawasaki disease in urban area with small Asian population. *American Journal of Diseases of Children*.

[28] Beiser AS, Takahashi M, Baker AL, Sundel RP, Newburger JW (1998). A predictive instrument for coronary artery aneurysms in Kawasaki disease. US Multicenter Kawasaki Disease Study Group. *American Journal of Cardiology*.

[29] Nakamura Y, Yashiro M, Uehara R (2003). Case-control study of giant coronary aneurysms due to Kawasaki disease. *Pediatrics International*.

[30] Harada K (1991). Intravenous gamma-globulin treatment in Kawasaki disease. *Acta Paediatrica Japonica*.

[31] Honkanen VE, McCrindle BW, Laxer RM, Feldman BM, Schneider R, Silverman ED (2003). Clinical relevance of the risk factors for coronary artery inflammation in Kawasaki disease. *Pediatric Cardiology*.

[32] Minich LL, Sleeper LA, Atz AM (2007). Delayed diagnosis of Kawasaki disease: what are the risk factors?. *Pediatrics*.

[33] Jennette JC (2002). Implications for pathogenesis of patterns of injury in small- and medium-sized-vessel vasculitis. *Cleveland Clinic Journal of Medicine*.

[34] Terai M, Kohno Y, Namba M (1990). Class II major histocompatibility antigen expression on coronary arterial endothelium in a patient with Kawasaki disease. *Human Pathology*.

[35] Burns JC, Glodé MP (2004). Kawasaki syndrome. *Lancet*.

[36] Ikari Y, McManus BM, Kenyon J, Schwartz SM (1999). Neonatal intima formation in the human coronary artery. *Arteriosclerosis, Thrombosis, and Vascular Biology*.

[37] Burns JC, Cayan DR, Tong G (2005). Seasonality and temporal clustering of Kawasaki syndrome. *Epidemiology*.

[38] Esper F, Shapiro ED, Weibel C, Ferguson D, Landry ML, Kahn JS (2005). Association between a novel human coronavirus and kawasaki disease. *Journal of Infectious Diseases*.

[39] Rowley AH, Baker SC, Shulman ST (2004). Detection of antigen in bronchial epithelium and macrophages in acute Kawasaki disease by use of synthetic antibody. *Journal of Infectious Diseases*.

